# Analysis of quality-adjusted survival time without symptoms or toxicity for pembrolizumab plus chemotherapy as treatment for previously untreated participants with advanced or metastatic esophageal cancer

**DOI:** 10.1007/s11136-025-04109-4

**Published:** 2026-02-01

**Authors:** Ying Zhang, Marc Diez Garcia, Sukrut Shah, Seongjung Joo, Adriana Valderrama, Shujing Zhang, Peter C. Enzinger

**Affiliations:** 1 MSD Europe Inc., Boulevard du Souverain 25, Brussels, 1170 Belgium; 2 Institut Català d´Oncologia – Campus Salut Bellvitge Comprehensive Cancer Center, L´Hospitalet de Llobregat, Barcelona, Spain; 3 Merck & Co., Inc., Rahway, NJ USA; 4 Dana-Farber Cancer Institute, Boston, MA USA

**Keywords:** Chemotherapy, Esophageal cancer, Health-related quality of life, Pembrolizumab

## Abstract

**Background:**

Results of the KEYNOTE-590 trial showed that first-line pembrolizumab plus chemotherapy significantly improved overall and progression-free survival versus chemotherapy alone, and the safety profile was manageable for participants with previously untreated advanced or metastatic esophageal cancer. Using the quality-adjusted time without symptoms or toxicity (Q-TWiST) method of analysis, we assessed the benefit/risk profile of pembrolizumab plus chemotherapy.

**Methods:**

Using data from the KEYNOTE-590 study, we partitioned participant survival time into three health states: time living with all-cause grade ≥ 3 adverse events (AEs) before disease progression (PD; TOX), time before start of PD or death without grade ≥ 3 AEs (TWiST), and time from the start of PD to death or the censoring date (REL). We calculated Q-TWiST by summing the restricted mean time spent in each health state weighted by health state utilities estimated using the EuroQol 5-Dimension, 5-Level quality-of-life questionnaire (EQ-5D-5L). The relative gain in quality-adjusted survival time was defined as the Q-TWiST difference divided by the survival time from chemotherapy alone. A relative gain of > 10% is considered “clinically important,” and a relative gain of > 15% is considered “clearly clinically important.” This analysis was primarily focused on clinical significance rather than statistical significance due to the nature of the Q-TWiST analyses. No prespecified formal hypothesis testing was performed, and hence, there was no adjustment for multiplicity.

**Results:**

At a maximum follow-up of 30 months, Q-TWiST was 2.23 months longer with pembrolizumab plus chemotherapy versus chemotherapy alone for all randomly assigned participants and was clearly clinically important, with a relative Q-TWiST gain of 17.86%. In all three subpopulations, including participants with esophageal squamous cell carcinoma (ESCC), programmed cell death ligand 1 (PD-L1) combined positive score (CPS) ≥ 10, and ESCC PD-L1 CPS ≥ 10, Q-TWiST gain with pembrolizumab plus chemotherapy versus chemotherapy was 2.29 to 3.87 months, equivalent to a relative Q-TWiST gain of 18.12% to 33.47%, which are all clearly clinically important.

**Conclusions:**

Although this analysis is limited by missing data and short follow-up time, pembrolizumab plus chemotherapy provided clinically meaningful and substantial benefit in quality-adjusted survival by Q-TWiST analysis versus chemotherapy alone in participants with advanced esophageal cancer.

**Trial registration:**

Trial registration for KEYNOTE-590 ClinicalTrials.gov, NCT03189719 (date of registration: June 14, 2017).

**Supplementary Information:**

The online version contains supplementary material available at 10.1007/s11136-025-04109-4.

## Introduction

Esophageal cancer is the sixth-leading cause of death from cancer worldwide, with a 5-year relative survival rate of approximately 25% [1, 2]. For several decades, the standard-of-care for untreated locally advanced or metastatic esophageal cancer has been chemotherapy combined with a fluoropyrimidine (fluorouracil or capecitabine) and a platinum agent (oxaliplatin or cisplatin), but overall survival (OS) remains poor [3]. Treatments that prolong OS for patients who have advanced esophageal cancer are necessary.

The KEYNOTE-590 (NCT03189719) trial was the first randomized phase 3 trial to show clinically meaningful and statistically significant improvement in OS (hazard ratio [HR], 0.73; 95% CI, 0.62–0.86; *p* < 0.0001) and progression-free survival (PFS; HR, 0.65, 95% CI, 0.55–0.76; *p* < 0.0001) with use of a programmed cell death protein 1 (PD-1) inhibitor combined with chemotherapy, compared with chemotherapy alone, in participants with previously untreated advanced esophageal cancer and Siewert type 1 gastroesophageal junction (GEJ) cancer [4]. A manageable safety profile was observed for pembrolizumab combined with chemotherapy compared with chemotherapy alone, and health-related quality of life (HRQOL) was maintained over time and was similar in the two treatment groups [4, 5].

Increasingly, regulatory bodies are demanding evaluation of the effect of a treatment on patient quality of life (QOL) [6]. The American Society of Clinical Oncology framework is focused on a net health benefit that measures the impact of an intervention by subtracting the division of incremental cost by the opportunity cost threshold from the incremental quality-adjusted life year gains. The Q-TWiST method is also used to assess treatment by integrating survival, toxicity, and QOL into a single measure, and it provides an analysis comparable to a net health benefit. With its relative simplicity and clinical relevance, Q-TWiST can better inform the shared decision making between clinicians and patients to assess the benefit of a treatment [7]. Analysis using the Q-TWiST method was initially adopted for adjuvant therapies in oncology, and it was widely used across different types of cancer over the past years [7–11]. For instance, Q-TWiST was used to evaluate the benefits of pazopanib versus sunitinib in patients with renal cell carcinoma, demonstrating a slightly longer Q-TWiST, primarily due to a reduced length of time with grade 3 or 4 toxicity [11]. This type of analysis complements traditional outcomes generated from clinical trials by providing evidence used to evaluate the benefit/risk profile for patients. We performed a Q-TWiST analysis using data from the KEYNOTE-590 study to examine the benefit/risk profile of adding pembrolizumab to standard-of-care chemotherapy for participants who have advanced esophageal cancer. To our knowledge, this is the first Q-TWiST study conducted for pembrolizumab plus chemotherapy in advanced esophageal cancer across different histologies.

## Materials and methods

### Data source and study population

We used participant-level data from KEYNOTE 590 (database cutoff, July 2, 2020) in this post hoc analysis. KEYNOTE 590 was a randomized, placebo-controlled, global, phase 3 trial of participants who had previously untreated, histologically or cytologically confirmed, locally advanced, unresectable or metastatic esophageal adenocarcinoma or esophageal squamous cell carcinoma (ESCC) or Siewert type 1 GEJ adenocarcinoma [4]. Participants were randomly assigned (1:1) to receive intravenous pembrolizumab 200 mg or placebo, both given at the same time as chemotherapy (5-fluorouracil 800 mg/m² on days 1–5 plus cisplatin 80 mg/m² on day 1, for a maximum of six cycles), once every 3 weeks for up to 35 cycles [4]. We used Q-TWIST for analysis of all randomly assigned participants, participants who had programmed cell death ligand 1 (PD-L1) combined positive score (CPS) ≥ 10 tumors, participants who had ESCC, and participants who had ESCC and a PD-L1 CPS ≥ 10.

The institutional review board or ethics committee at each participating institution approved the trial protocol and all amendments. The study was conducted in accordance with principles of Good Clinical Practice and was approved by the appropriate institutional review boards and regulatory agencies. All participants provided written informed consent before enrollment.

### Assessments

OS was defined as the time from randomization to the date of death from any cause. PFS was defined as the time from random assignment of the participant to documented beginning of disease progression per Response Evaluation Criteria in Solid Tumors, version 1.1 (RECIST v1.1), according to the investigator, or as death from any cause. Tumor response was assessed per RECIST v1.1 according to the investigator.

We evaluated adverse events (AEs) throughout the trial until 30 days (90 days for serious AEs) after the end of treatment, per the National Cancer Institute Common Terminology Criteria for Adverse Events, version 4.0. We evaluated QoL using EuroQol 5-Dimension, 5-Level Quality-of-Life questionnaire (EQ-5D-5-L) scores.

### Statistical analysis

For the Q-TWiST analysis, we grouped survival time into three health states:

1. Toxicity (TOX), defined as time with all-cause grade ≥ 3 AEs between randomization date and disease progression date or PFS censoring date. We calculated the duration of grade ≥ 3 AEs as the time between the AE onset date and the AE resolution date for AEs that had resolved by the time of disease progression, or the date of disease progression for ongoing AEs at the time of disease progression. Participants who did not experience grade ≥ 3 AEs before disease progression were assigned a duration of zero. The duration of an AE was considered the entire duration of any AE episode that started as or worsened to grade ≥ 3. The total number of days with all-cause grade ≥ 3 AEs was combined for each participant, where a day with multiple events was only counted once.
2. Time without symptoms and toxicity (TWiST), defined as time from randomization to the beginning of disease progression per RECIST v1.1 according to investigator assessment, to death, or to censoring date of PFS without all-cause grade ≥ 3 AEs.
3. Relapse (REL), defined as time from the start of progressive disease to death or censoring date (last follow-up or database cutoff) [8, 9].

The Kaplan-Meier method was used to estimate OS, PFS, and TOX in both treatment groups. The mean duration spent in each health state (TOX, TWiST, and REL) was calculated as the restricted mean survival time [12] from time 0 to month 30 (maximum follow-up).

We calculated the mean duration of the TWiST and REL health states as follows:

$$\mathrm{T}\mathrm{W}\mathrm{i}\mathrm{S}\mathrm{T}\:=\mathrm{P}\mathrm{F}\mathrm{S}-\mathrm{T}\mathrm{O}\mathrm{X}$$

and

$$\mathrm{R}\mathrm{E}\mathrm{L}\:=\mathrm{O}\mathrm{S}-\mathrm{P}\mathrm{F}\mathrm{S}$$

EQ-5D-5-L translates five dimensions of QOL (mobility, self-care, usual activities, pain/discomfort, and anxiety/depression) into a single utility score. Postbaseline pooled EQ-5D-5-L scores collected in KEYNOTE-590 were used to derive average utility weights for each health state (Utility_TOX_, Utility_TWiST_, and Utility_REL_) using the US mapping algorithm [13]. The US-specific mapping algorithm was selected due to its widespread use and validation in oncology health utility analyses, providing a consistent and comparable framework for interpreting quality of life outcomes. Furthermore, the US tariff offers a standardized reference that aligns with regulatory expectations and facilitates comparison with other studies using similar methodology.

We calculated Q-TWiST as the sum of the restricted mean time spent in each health state, weighted by its corresponding health state utility using the following formula:

$$ \begin{aligned} {\mathrm{Q-TWiST}} = & \left( {{\mathrm{TOX}}\: \times \:{\mathrm{Utility}}_{{{\mathrm{TOX}}}} } \right) \\ & \quad + \left( {{\mathrm{TWiST}}\: \times \:\:{\mathrm{Utility}}_{{{\mathrm{TWiST}}}} } \right) \\ & \quad + \left( {{\mathrm{REL}}\: \times \:\:{\mathrm{Utility}}_{{{\mathrm{REL}}}} } \right) \\ \end{aligned} $$

Differences in restricted mean time spent in each health state and in Q-TWiST between treatment groups were calculated. The corresponding 95% CIs were generated using bootstrap sampling stratified by treatment groups. Relative Q-TWiST gain was calculated as the Q-TWiST difference (defined as difference between Q-TWiST scores of the two treatment groups) between the pembrolizumab plus chemotherapy group and chemotherapy alone group divided by the restricted mean OS of the chemotherapy group. No prespecified formal hypothesis testing was performed, and hence, there was no adjustment for multiplicity.

We conducted three sensitivity analyses, calculated the mean Q-TWiST difference and relative gain at different time points, and plotted to assess the Q-TWiST results over time. To explore the robustness of the TOX health state, we performed a sensitivity analysis considering all-cause AE regardless of grade. Two sensitivity analyses were carried out to account for the impact of missing EQ-5D-5-L data (especially post-treatment discontinuation, in the REL state) and assess the robustness of the Q-TWiST results to various assumptions about the utility values: (1) base case utility values where Utility_TOX_ and Utility_REL_ were set equal to 0.5 and Utility_TWiST_ equal to 1.0, which is commonly used in many Q-TWiST publications and enables a straightforward comparison of results across studies for clinical decision-making [11, 14–16] and (2) threshold analysis, particularly addressing the missing EQ-5D-5-L data in TOX and REL health states, where Utility_TOX_ and Utility_REL_ weights varied from 0 to 1 simultaneously at increments of 0.1, while holding Utility_TWiST_ equal to 1 (representing perfect health).

The relative gain in quality-adjusted survival time was defined as “clinically important” or “clearly clinically important,” respectively, on the basis of published thresholds in the literature, where clinically important is representative of a gain ≥ 10% and “clearly clinically important” is representative of a gain ≥ 15% [7].

## Results

### Participants

In the KEYNOTE-590 trial, 749 participants were enrolled, of which 373 were randomly assigned to receive pembrolizumab plus chemotherapy and 376 to receive chemotherapy alone. Participant baseline characteristics were previously published [4]; the characteristics were well balanced between treatment groups [4]. Participant completion and compliance rates for the EQ-5D-5L are shown in Online Resource Table S1.

## Duration of TOX, TWiST, and REL states

**Fig. 1:**
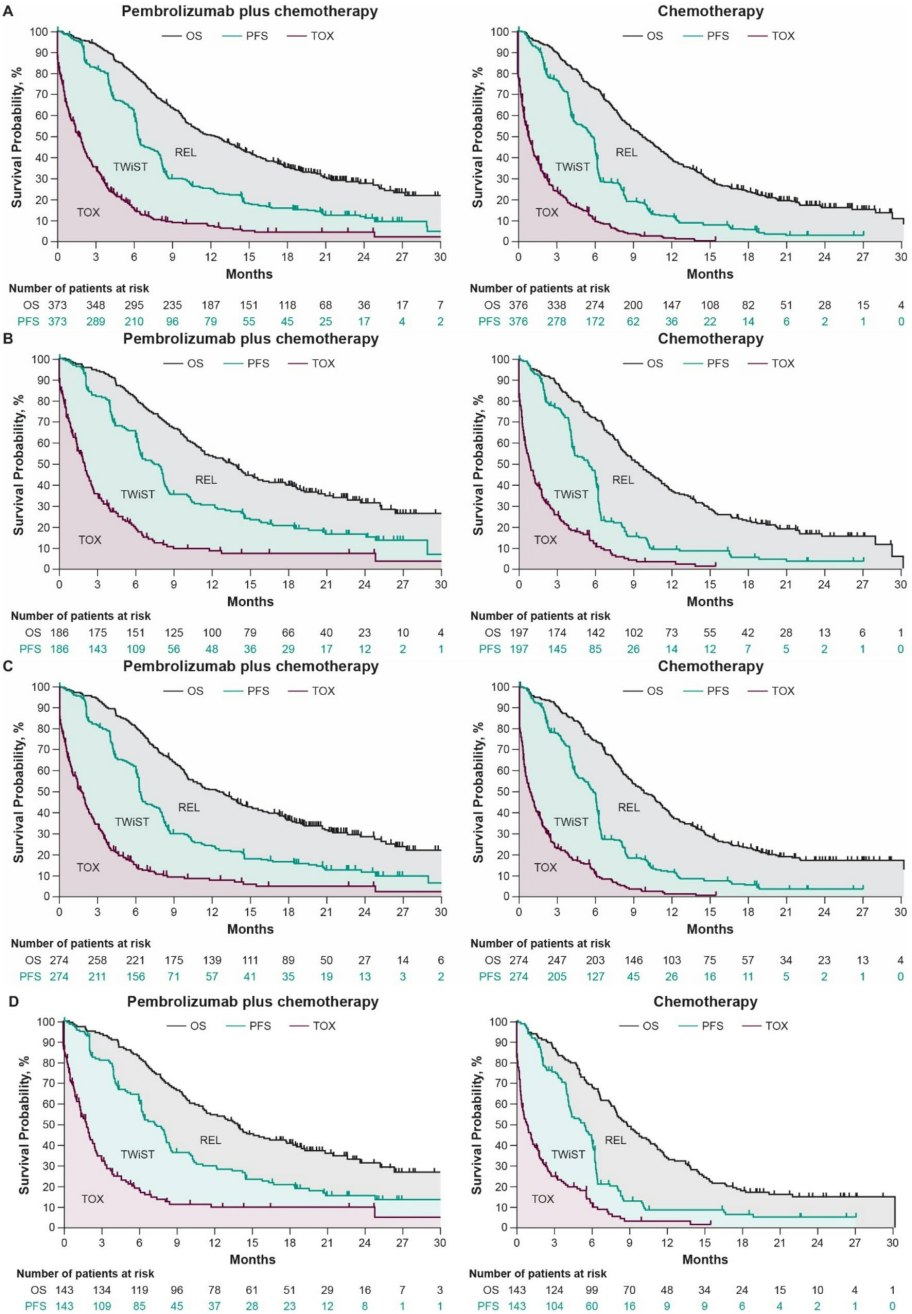
Partitioned Kaplan-Meier survival curves for the Q-TWiST health states in **A** all randomly assigned participants and participants with **B** PD-L1 CPS ≥ 10 tumors, **C** ESCC, and **D** ESCC and a PD-L1 CPS ≥ 10. *CPS* combined positive score, *ESCC* esophageal squamous cell carcinoma, *OS* overall survival, *PD-L1* programmed cell death ligand 1, *PFS* progression-free survival, *REL* relapse, *TOX* toxicity, *TWiST* time without symptoms or toxicity

Using the Kaplan-Meier curve for OS, PFS, and TOX for each treatment group separately, the time spent in each of the three Q-TWiST health states is illustrated by the shaded area between the curves across four populations (Fig. 1).

At month 30, the mean duration was longer in the TOX state (1.57 months; 95% CI, 0.76–2.39) and the TWiST state (1.39 months; 95% CI, 0.04–2.84) and shorter in the REL state (0.30 month; 95% CI, − 2.07 to 1.38) for all randomly assigned participants in the pembrolizumab plus chemotherapy group compared with the chemotherapy alone group (Table 1).

**Table 1:** Restricted mean duration in the three different health states and Q-TWiST difference at month 30 for the intention-to-treat population

	Pembrolizumab plus chemotherapy	Chemotherapy	Difference (95% CI^a^), months
**All randomly assigned participants**, ***N***	373	376	NA
TOX	3.69	2.12	1.57 (0.76–2.39)
TWiST	5.83	4.44	1.39 (0.04–2.84)
REL	5.65	5.95	−0.30 (− 2.07 to 1.38)
Q-TWiST^b^	11.98	9.75	2.23 (1.25–3.26)
Q-TWiST^c^	10.50	8.48	2.02 (1.10–3.04)
**PD-L1 CPS ≥ 10,** *** n***	186	197	NA
TOX	4.37	2.23	2.14 (0.81–3.46)
TWiST	6.32	4.11	2.20 (0.07–4.41)
REL	5.42	5.79	−0.37 (− 2.87 to 2.33)
Q-TWiST^b^	12.82	9.45	3.37 (1.97–4.87)
Q-TWiST^c^	11.21	8.12	3.09 (1.68–4.51)
**ESCC**, ***n***	274	274	NA
TOX	3.76	2.12	1.64 (0.71–2.64)
TWiST	5.78	4.49	1.29 (− 0.26 to 2.93)
REL	5.82	6.02	−0.20 (− 2.31 to 1.91)
Q-TWiST^b^	12.17	9.88	2.29 (1.12–3.48)
Q-TWiST^c^	10.58	8.56	2.01 (0.86–3.12)
**ESCC PD-L1 CPS ≥ 10,** *** n***	143	143	NA
TOX	4.71	2.27	2.44 (0.76–4.21)
TWiST	5.97	4.05	1.92 (− 0.60 to 4.22)
REL	5.56	5.24	0.31 (− 2.43 to 3.41)
Q-TWiST^b, c^	13.01	9.13	3.87 (2.24–5.55)
Q-TWiST^b, d^	11.11	7.81	3.30 (1.53–5.04)

Data are expressed in months except as noted otherwise.

*CPS* combined positive score, *ESCC* esophageal squamous cell carcinoma, *EQ-5D-5L *EuroQol 5-dimension, 5-level questionnaire, *NA* not applicable, *PD-L1* programmed cell death ligand 1, *Q-TWiST* quality-adjusted time without symptoms of disease progression or toxicity of treatment, REL relapse, *TOX* toxicity, *TWiST* time without symptoms or toxicity

^a^Based on bootstrap percentiles. ^b^Both pooled-utility and standardized-weight results are shown to offer complementary perspectives on the Q-TWiST analysis. Pooled-utility values reflect the actual patient-reported utility scores observed in the study population. In contrast, standardized weights apply fixed utility values to each health state, allowing for easier comparison across studies and reducing the influence of sample-specific utility distributions. ^c^Based on the US mapping algorithm–derived mean EQ-5D-5L health utility weights for the pooled population (see Table 2). ^d^Standardized utility weights are based on 0.5 for Utility_TOX_, 1.0 for Utility_TWiST_, and 0.5 for Utility_REL_.

We observed a similar trend in the participants with PD-L1 CPS ≥ 10 tumors and participants with ESCC. Notably, the mean duration of all three health states was longer in the participants with ESCC and a PD-L1 CPS ≥ 10 for the pembrolizumab plus chemotherapy group than in the chemotherapy alone group (Table 1).

## Q-TWiST

**Fig. 2:**
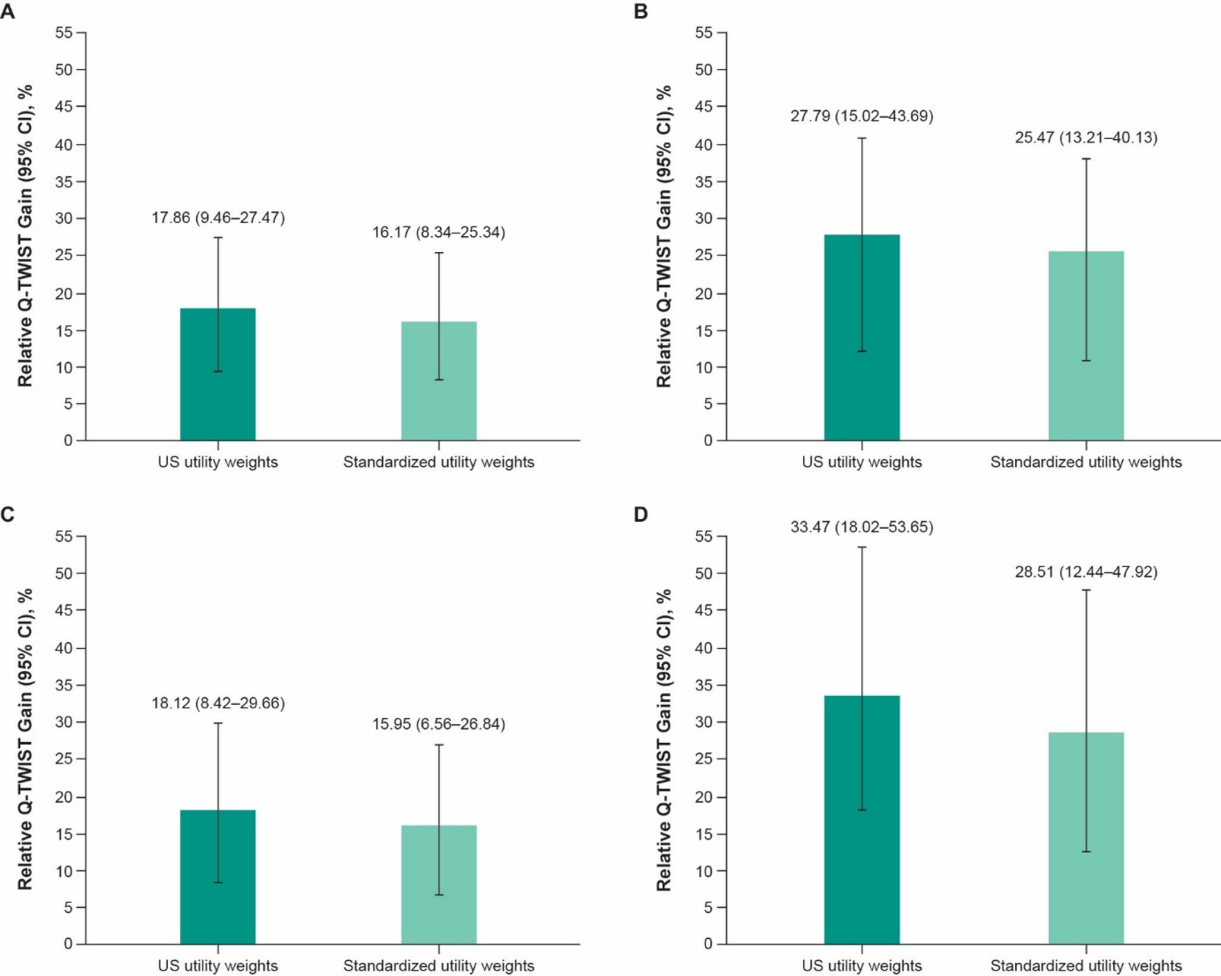
Relative Q-TWiST gain for pembrolizumab plus chemotherapy compared with chemotherapy alone in **A** all randomly assigned participants and participants with **B** PD-L1 CPS ≥ 10 tumors, **C** ESCC, and **D** ESCC and a PD-L1 CPS ≥ 10. US utility weights were derived from postbaseline EQ-5D-5L scores using US mapping algorithm. Standardized utility weights are based on 0.5 for Utility_TOX_, 1.0 for Utility_TWiST_, and 0.5 for Utility_REL_. *CPS* combined positive score, *ESCC* esophageal squamous cell carcinoma, *EQ-5D-5L* EuroQol 5-dimension, 5 level questionnaire, *PD-L1* programmed cell death ligand 1, *Q-TWiST* quality-adjusted time without symptoms of disease progression or toxicity of treatment, *Utility*_*REL*_ utility of the REL state, *Utility*_*TOX*_ utility of the TOX state, *Utility*_*TWiST*_ utility of the TWiST state

Table 1 shows the restricted mean duration in the three different health states and restricted mean Q-TWiST at month 30 for the intention-to-treat population. After applying the pooled mean US health utility weights for each health state (Table 2), we saw that the Q-TWiST difference favored pembrolizumab plus chemotherapy compared with chemotherapy alone by 2.23 months for all randomly assigned participants (relative gain, 17.86%; Table 1; Fig. 2A).

**Table 2:** Pooled^a^ mean EQ-5D-5 L health utility weights using the US mapping algorithm

	*n* ^b^	Utility weights^c^	
		Mean	95% CI
All randomly assigned participants (*N* = 727)			
TOX	402	0.792	0.780–0.804
TWiST	546	0.868	0.861–0.874
REL	413	0.708	0.687–0.729
**PD-L1 CPS ≥ 10 (*n*** **= 372)**			
TOX	209	0.797	0.781–0.813
TWiST	273	0.874	0.866–0.883
REL	210	0.704	0.675–0.732
**ESCC (*n*** **= 532)**			
TOX	297	0.794	0.779–0.808
TWiST	402	0.872	0.865–0.880
REL	318	0.711	0.686–0.735
**ESCC PD-L1 CPS ≥ 10 (** ***n*** ** = 278)**			
TOX	156	0.803	0.785–0.820
TWiST	204	0.878	0.868–0.888
REL	159	0.716	0.683–0.750

*CPS* combined positive score, *ESCC* esophageal squamous cell carcinoma, *EQ-5D-5L* EuroQol 5-dimension, 5-level questionnaire, *PD-L1* programmed cell death ligand 1, *TWiST* time without symptoms or toxicity, *REL* relapse, *TOX* toxicity.

^a^Both treatment groups*. *^b^Number of participants with at least one nonmissing EQ-5D-5L score. ^c^Summary statistics computed based on several records per participant treated as independent observations, except for baseline, for which there is a single record per participant.

We saw a similar trend in each subpopulation (PD-L1 CPS ≥ 10 tumors: 3.37 months [relative gain, 27.79%; Table 1; Fig. 2B]; ESCC: 2.29 months [relative gain, 18.12%; Table 1; Fig. 2C]; ESCC and a PD-L1 CPS ≥ 10: 3.87 months [relative gain, 33.47%; Table 1; Fig. 2D]).

### Sensitivity analyses

For all randomly assigned participants across all three subpopulations, the participants treated with pembrolizumab plus chemotherapy showed a positive mean Q-TWiST difference and relative Q-TWiST gain compared with chemotherapy alone starting at month 9; the benefit increased with longer duration of follow-up (Online Resource Fig. S1). In the analysis with standardized utility weights (0.5 for TOX, 1.0 for TWiST, and 0.5 for REL), mean Q-TWiST was 2.02 months longer for all randomly assigned participants (relative gain, 16.17%; Table 1; Fig. 2A) for pembrolizumab plus chemotherapy compared with chemotherapy alone. We saw a similar trend in all three subpopulations (participant with PD-L1 CPS ≥ 10 tumors: 3.09 months [relative gain, 25.47%; Table 1; Fig. 2B]; participants who had ESCC: 2.01 months [relative gain, 15.95%; Table 1; Fig. 2C]; participants who had ESCC and a PD-L1 CPS ≥ 10: 3.30 months [relative gain, 28.51%; Table 1; Fig. 2D]).

**Fig. 3:**
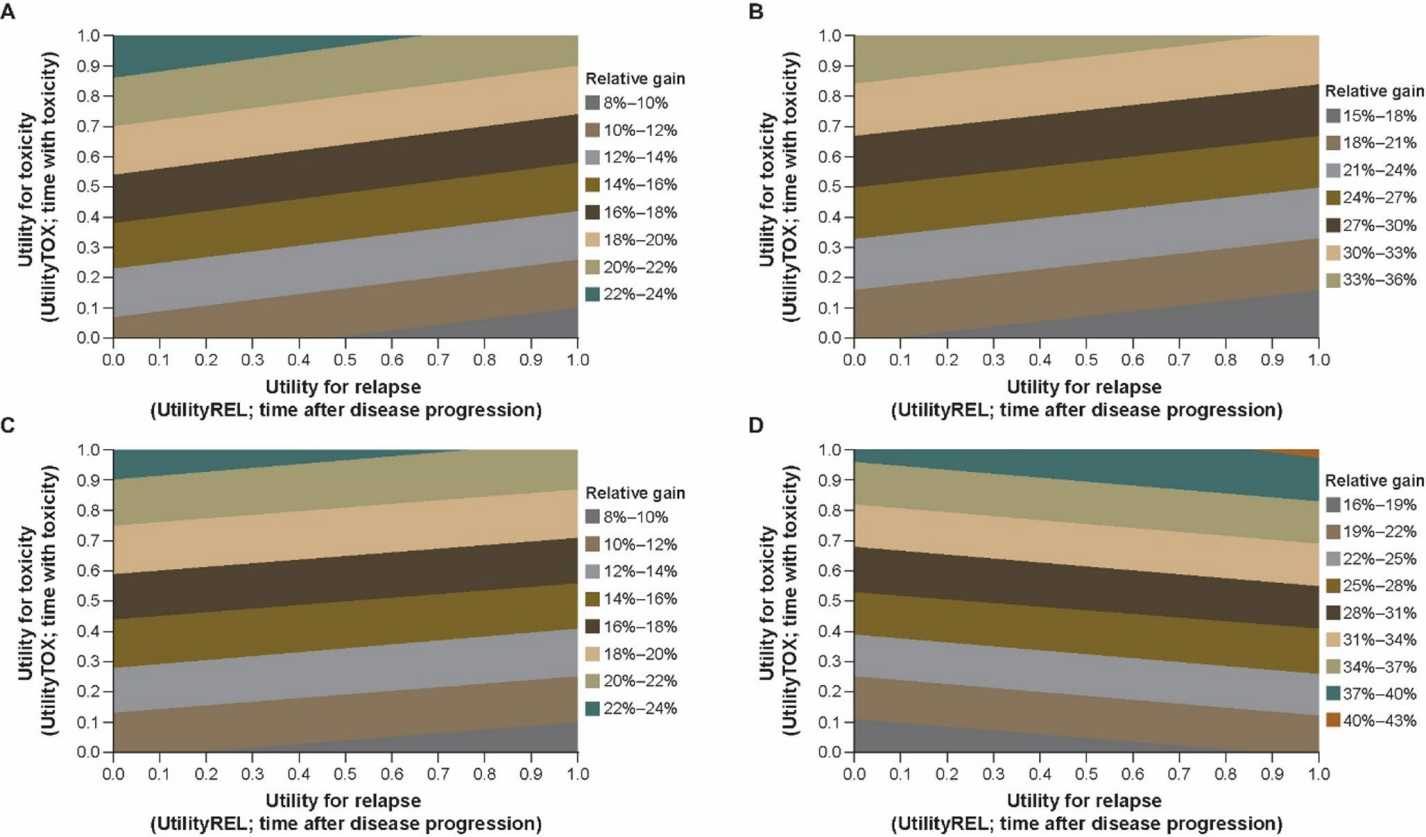
Threshold utility analysis of Q-TWiST with a maximum follow-up of 30 months for **A** all randomly assigned participants and participants with **B** PD-L1 CPS ≥ 10 tumors, **C** ESCC, and **D** ESCC and a PD-L1 CPS ≥ 10. Utility_TOX_ and Utility_REL_ were varied from 0 to 1 by increments of 0.01 while Utility_TWiST_ was kept constant at 1.0 for all Q-TWiST calculations. *CPS* combined positive score, *ESCC* esophageal squamous cell carcinoma, *PD-L1* programmed cell death ligand 1, *Utility*_*REL*_ utility of the REL state, *Utility*_*TOX*_ utility of the TOX state, *Utility*_*TWiST*_ utility of the TWiST state, *Q-TWiST* quality-adjusted time without symptoms of disease progression or toxicity of treatment

Noting that Utility_TOX_ and Utility_REL_ simultaneously varied between 0.00 and 1.00 at increments of 0.1, the mean Q-TWiST difference for pembrolizumab plus chemotherapy compared with chemotherapy alone increased from 1.09 to 2.96 months (relative gain, 8.68% to 23.65%) for all randomly assigned participants (Fig. 3A). We observed a similar trend in all three subpopulations (participants with PD-L1 CPS ≥ 10 tumors: increased from 1.84 to 4.34 months [relative gain, 15.15% to 35.78%; Fig. 3B]; participants with ESCC: increased from 1.09 to 2.94 months [relative gain, 8.66% to 23.23%; Fig. 3C]; participants who had ESCC and a PD-L1 CPS ≥ 10: increased from 1.92 to 4.68 months [relative gain, 16.59% to 40.43%; Fig. 3D]).

In the sensitivity analysis for the TOX state when the definition of TOX was changed to time with all-cause AEs regardless of grade, the mean Q-TWiST difference for pembrolizumab plus chemotherapy versus chemotherapy alone decreased from 2.23 to 1.32 months at month 30 for all randomly assigned participants (relative gain, 17.86% to 10.56%; Online Resource Table S2). We observed a similar trend in the participants with PD-L1 CPS ≥ 10 tumors subpopulation (decreased from 3.37 to 1.97 months [relative gain, 27.79% to 16.28%]) and the participant who had ESCC subpopulation (decreased from 2.29 to 1.39 months [relative gain, 18.12% to 11.02%]) (Online Resource Table S2). In the participants who has ESCC and a PD-L1 CPS ≥ 10 subpopulation, an increase from 3.87 to 4.13 months (relative gain, 33.47% to 35.69%) was observed (Online Resource Table S2).

## Discussion

In KEYNOTE-590, OS and PFS were better for participants treated with pembrolizumab plus chemotherapy compared with those who received only chemotherapy. However, use of pembrolizumab plus chemotherapy also led to increased toxicity. Thus, when evaluating treatment options, clinical efficacy, toxicity, and HRQoL should be considered.

Q-TWiST analyses are generally focused on clinical significance rather than on statistical significance, reporting effect sizes and the corresponding 95% CIs. In the current analysis, the mean difference and relative gain in quality-adjusted survival time between treatment groups were clinically meaningful and were substantial for all randomly assigned participants across the three subpopulations evaluated. The gain in quality-adjusted survival time was primarily due to a longer PFS duration. The numerically longer mean Q-TWiST observed for pembrolizumab plus chemotherapy versus for chemotherapy alone at month 30 supports the OS and PFS data observed in the KEYNOTE-590 trial [4], as well as observations from HRQoL analyses in which pembrolizumab plus chemotherapy did not negatively impact QoL compared with chemotherapy alone [5]. The 2.23 month gain in Q-TWiST indicates that patients may experience over two additional months of quality-adjusted and symptom-free survival compared to the control arm. In clinical practice, this translates to a meaningful extension of time during which patients are likely to remain symptom free and toxicity free, supporting continued functional independence and engagement in daily activities, potentially delaying transitions to more intensive care, and thereby helping clinicians and patients make more informed treatment decisions.

The relative Q-TWiST gain in the current analysis was 17.86% at a maximum follow-up of 30 months for all randomly assigned participants, a numerical improvement over the relative Q-TWiST gain (13%) reported for nivolumab plus chemotherapy versus chemotherapy alone at a maximum follow-up of 33 months for previously untreated participants with advanced gastric cancer, GEJ cancer, or esophageal adenocarcinoma [17]. In participants with ESCC, nivolumab plus ipilimumab and nivolumab plus chemotherapy both provided clinically meaningful improvement in Q-TWiST compared with chemotherapy alone (5.7 weeks and 7.6 weeks, respectively) after a minimum follow-up of 29 months [16]. The relative gain in quality-adjusted survival time of more than 15% is consistent with the relative gain of 17.0% to 20.0% reported for pembrolizumab monotherapy or in combination with chemotherapy compared with chemotherapy alone in participants with different tumor types [15, 18], and it is higher than the relative Q-TWiST gain reported across tumors and therapies in a previous systematic literature review [14]. In the systematic literature review that contextualizes and benchmarks Q-TWiST analysis [14], 51 articles with 81 distinct Q-TWiST comparisons were identified. As reported, the mean (median) relative Q-TWiST gains were 7.8% (7.2%) across all cancer types. Forty percent of the studies reported a relative Q-TWiST gain ≥ 10% (defined as clinically important difference) and 22.7% of the studies reported a relative Q-TWiST gain ≥ 15% (defined as clearly clinically important difference). The 10% and 15% thresholds were recommended in a previous systematic literature review conducted by Revicki, Feeny, Hunt, and Cole [7]. However, the authors stated that these clinically important difference estimates should be interpreted with caution, pending confirmation in future studies by direct patient assessment of the clinically relevant health states for Q-TWiST. Additionally, one should make comparisons to the literature with caution, considering the differences in participant baseline characteristics, follow-up duration, tumor type, and treatment regimen. The relative gain in quality-adjusted survival time was more pronounced in participants with PD-L1 CPS ≥ 10 tumors and participants who had ESCC and a PD-L1 CPS ≥ 10, likely a result of the extended PFS benefit observed. In addition, the relative gain in quality-adjusted survival time obtained in the base case exceeded established thresholds reported in the literature of at least 15.0%, which is considered “clearly clinically important,” although these established thresholds are not specific to participants with advanced esophageal cancer [7]. With a relative gain of greater than 15%, pembrolizumab plus chemotherapy conferred a clearly clinically meaningful improvement in quality-adjusted survival time compared with chemotherapy alone. Q-TWiST analysis provides an assessment of the benefit–risk associated with cancer treatment. These findings continue to support the use of pembrolizumab plus chemotherapy for this population.

As observed from the sensitivity analyses, the relative gain in quality-adjusted survival time increased as follow-up time increased, indicating a better PFS benefit. Using standardized utilities as mentioned in the methods section, a lower relative gain in quality-adjusted survival time was observed compared with using US utilities, implying that the impact of combined TOX and REL outweighed the impact of TWiST. The utility threshold analysis showed that Utility_TOX_ had a more substantial impact on relative Q-TWiST gain than Utility_REL_, as expected given the larger difference in restricted time spent in the TOX state compared with the REL state. With increasing Utility_TOX_, the relative Q-TWiST gain increased across all three subpopulations, whereas the relative Q-TWiST gain increased with increasing Utility_REL_ for all subpopulations except ESCC PD-L1 CPS ≥ 10.

One of the limitations of the primary analysis is the TOX state only includes grade 3–5 AEs and ignores the potential impact of grade 1–2 AEs on QOL. To account for this limitation, we conducted a sensitivity analysis that defined TOX as grade 1–2 AEs. Additionally, post-progression toxicities were not included in the analysis and may also influence results. Furthermore, missing EQ-5D-5L data and the short follow-up time might introduce a potential bias when interpreting data. However, compliance with scheduled EQ-5D-5L assessments was high (approximately 90%) and well balanced between treatment arms. The utility threshold analysis, conducted to assess how different utility thresholds affect the estimated treatment effect, consistently demonstrated a benefit of pembrolizumab plus chemotherapy over chemotherapy alone. Immunotherapy is often associated with long-term survival and delayed treatment effects; therefore, with short follow-up times, using the traditional Q-TWiST method could contribute to underestimation of the quality-adjusted survival length with immunotherapies. Performing Q-TWiST analyses with longer follow-up times is expected to further reduce uncertainty. Short-term data may provide an incomplete assessment of the treatment effect; long-term data are needed to ensure the validity and reliability of these results. However, the sensitivity analysis showed the relevant treatment effect increased over time. Therefore, the Q-TWiST difference and relative gain would likely increase with longer follow-up.

## Conclusion

Results of the current Q-TWiST analysis showed that use of pembrolizumab plus chemotherapy caused clinically meaningful and substantial improvement in quality-adjusted survival compared with chemotherapy alone in participants with untreated locally advanced or metastatic esophageal cancer. The relative gain in quality-adjusted survival time exceeded the established thresholds in the literature, defined as either “clinically important” or “clearly clinically important.” The sensitivity analyses showed results were similar regardless of different follow-up periods, utility values, and definitions of the TOX state. The results of the current analysis add evidence from participants’ perspectives to strengthen the benefit/risk profile of pembrolizumab plus chemotherapy for the assessed population. Future studies with longer follow-up will be important to validate the current findings considering the delayed treatment effects often associated with immunotherapy.

## Supplementary Information

Below is the link to the electronic supplementary material.

Supplementary Material 1

## Data Availability

Merck Sharp & Dohme LLC, a subsidiary of Merck & Co., Inc., Rahway, NJ, USA (MSD) is committed to providing qualified scientific researchers access to anonymized data and clinical study reports from the company’s clinical trials for the purpose of conducting legitimate scientific research. MSD is also obligated to protect the rights and privacy of trial participants and, as such, has a procedure in place for evaluating and fulfilling requests for sharing company clinical trial data with qualified external scientific researchers. The MSD data sharing website (available at: http://externaldatasharing-msd.com/) outlines the process and requirements for submitting a data request. Applications will be promptly assessed for completeness and policy compliance. Feasible requests will be reviewed by a committee of MSD subject matter experts to assess the scientific validity of the request and the qualifications of the requestors. In line with data privacy legislation, submitters of approved requests must enter into a standard data-sharing agreement with MSD before data access is granted. Data will be made available for request after product approval in the United States and the European Union or after product development is discontinued. There are circumstances that may prevent MSD from sharing requested data, including country or region-specific regulations. If the request is declined, it will be communicated to the investigator. Access to genetic or exploratory biomarker data requires a detailed, hypothesis-driven statistical analysis plan that is collaboratively developed by the requestor and MSD subject matter experts; after approval of the statistical analysis plan and execution of a data-sharing agreement, MSD will either perform the proposed analyses and share the results with the requestor or will construct biomarker covariates and add them to a file with clinical data that is uploaded to an analysis portal so that the requestor can perform the proposed analyses.
