# Supplementary material for: Analysis of quality-adjusted survival time without symptoms or toxicity for pembrolizumab plus chemotherapy as treatment for previously untreated participants with advanced or metastatic esophageal cancer
Source: Qual Life Res. 2026 Feb 1;35(3):58. doi: 10.1007/s11136-025-04109-4 (PMC12861989; doi:10.1007/s11136-025-04109-4)
Supplement: Supplementary file 1 — Supplementary Material 1 [file 11136_2025_4109_MOESM1_ESM.docx]

**ONLINE RESOURCE**

**Fig. S1** Mean Q-TWiST gain based on the US health utility weight and relative Q-TWiST gain of pembrolizumab plus chemotherapy versus chemotherapy over time in (A) all randomly assigned participants and participants with (B) PD-L1 CPS ≥10 tumors, (C) ESCC, and (D) ESCC and a PD-L1 CPS ≥10. *CPS* combined positive score, *ESCC* esophageal squamous cell carcinoma, *PD-L1* programmed cell death ligand 1, *Q-TWiST* quality-adjusted time without symptoms of disease progression or toxicity of treatment


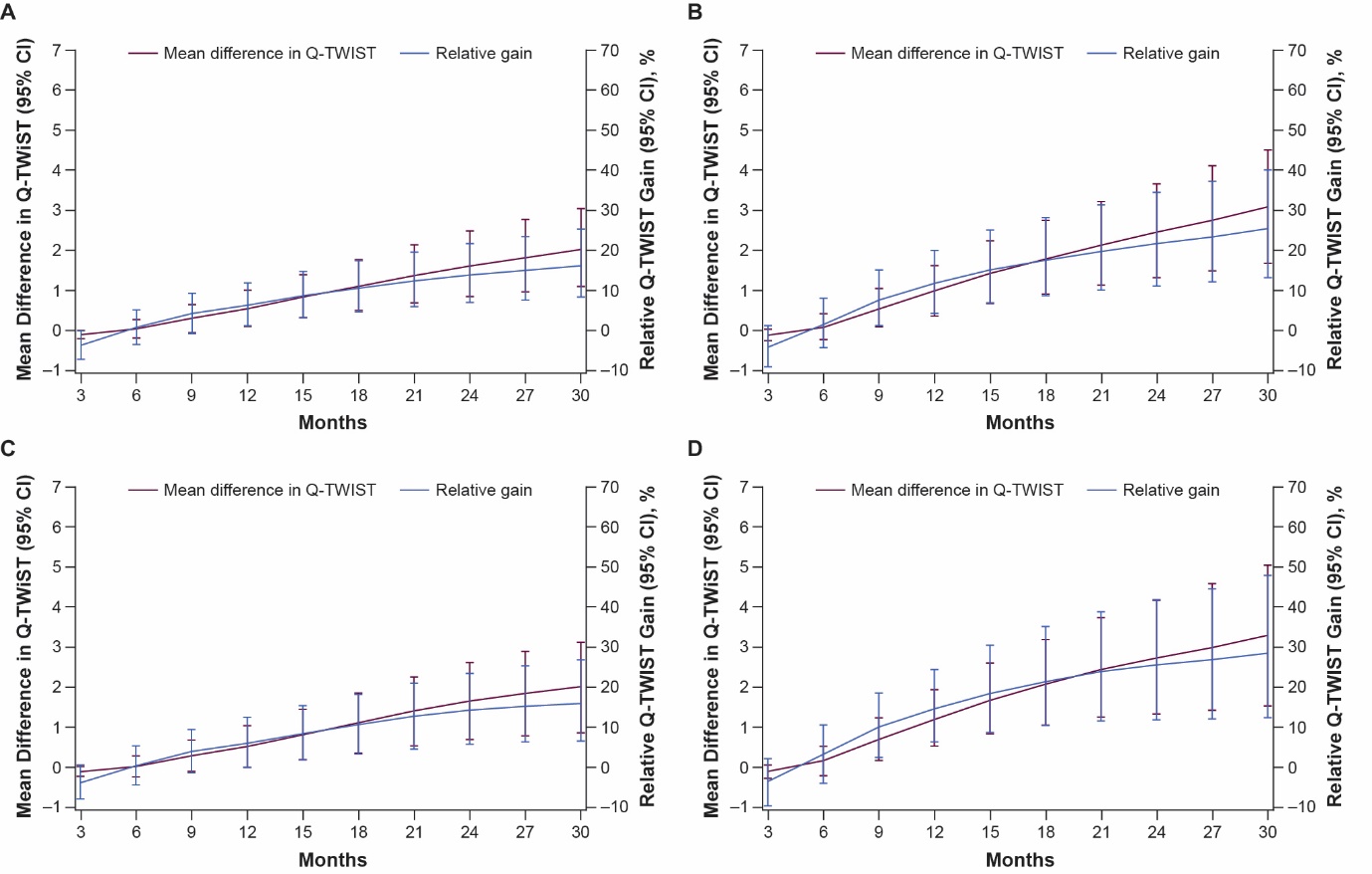


**Table S1** **Completion and compliance percentages for the EQ-5D-5L by visit and treatment in the full analysis set population^a^**

|  | **Pembrolizumab plus chemotherapy**  ***n* = 367** | **Chemotherapy**  ***n* = 360** |
| --- | --- | --- |
| Baseline |  |  |
| Completion | 360 (98.1) | 353 (98.1) |
| Compliance | 360 (98.1) | 353 (98.1) |
| Week 3 |  |  |
| Completion | 316 (86.1) | 292 (81.1) |
| Compliance | 316/330 (95.8) | 295/311 (93.9) |
| Week 6 |  |  |
| Completion | 290 (79.0) | 278 (77.2) |
| Compliance | 290/306 (94.8) | 278/293 (94.9) |
| Week 9 |  |  |
| Completion | 265 (72.2) | 278 (77.2) |
| Compliance | 265/281 (94.3) | 278/304 (91.4) |
| Week 12 |  |  |
| Completion | 256 (69.8) | 238 (66.1) |
| Compliance | 256/274 (93.4) | 238/265 (89.8) |
| Week 15 |  |  |
| Completion | 237 (64.6) | 225 (62.5) |
| Compliance | 237/258 (91.9) | 225/244 (92.2) |
| Week 18 |  |  |
| Completion | 226 (61.6) | 204 (56.7) |
| Compliance | 226/250 (90.4) | 204/220 (92.7) |
| Week 21 |  |  |
| Completion | 207 (56.4) | 189 (52.5) |
| Compliance | 207/220 (94.1) | 189/197 (95.5) |
| Week 24 |  |  |
| Completion | 208 (56.7) | 188 (52.2) |
| Compliance | 208/222 (93.7) | 188/201 (93.5) |
| Week 33 |  |  |
| Completion | 155 (42.2) | 126 (35.0) |
| Compliance | 155/183 (84.7) | 126/143 (88.1) |
| Week 42 |  |  |
| Completion | 109 (29.7) | 74 (20.6) |
| Compliance | 109/127 (85.8) | 74/88 (84.1) |
| Week 51 |  |  |
| Completion | 80 (21.8) | 42 (11.7) |
| Compliance | 80/90 (88.9) | 42/55 (76.4) |
| Week 60 |  |  |
| Completion | 31 (8.4) | 26 (7.2) |
| Compliance | 31/78 (39.7) | 26/41 (63.4) |
| Week 69 |  |  |
| Completion | 10 (2.7) | 3 (0.8) |
| Compliance | 10/68 (14.7) | 3/22 (13.6) |
| Week 78 |  |  |
| Completion | 4 (1.1) | 4 (1.1) |
| Compliance | 4/54 (7.4) | 4/16 (25.0) |
| Week 87 |  |  |
| Completion | 6 (1.6) | 1 (0.3) |
| Compliance | 6/44 (13.6) | 1/9 (11.1) |
| Week 96 |  |  |
| Completion | 0 (0) | 0 (0) |
| Compliance | 0/32 (0) | 0/3 (0) |
| Week 105 |  |  |
| Completion | 13 (3.5) | 0 (0) |
| Compliance | 13/22 (59.1) | 0/2 (0) |
| Week 114 |  |  |
| Completion | 8 (2.2) | 0 (0) |
| Compliance | 8/8 (100) | 0/1 (0) |

Data are *n* (%) unless otherwise noted

*EQ-5D-5L* EuroQol 5-dimension, 5-level questionnaire

^a^Compliance is defined as the proportion of participants who completed the participant-reported outcomes questionnaire among those who were expected to complete the questionnaire at the specified time point, excluding those missing by design (due to adverse event, death, discontinuation, translations not available, or no visit scheduled)

**Table S2 Sensitivity analysis with toxicity as all-cause AEs**

|  | **Pembrolizumab plus chemotherapy** | **Chemotherapy** | **Difference (95% CI), months** | **Relative Q-TWiST (95% CI), %** |
| --- | --- | --- | --- | --- |
| **ITT, *n*** | 373 | 376 | NA | NA |
| 12 months | 4.85 | 4.55 | 0.31 (−0.12 to 0.76) | 3.57 (−1.37 to 8.86) |
| 30 months | 7.87 | 6.55 | 1.32 (0.32–2.37) | 10.56 (2.47–19.40) |
| **PD-L1 CPS ≥10, *n*** | 186 | 197 | NA | NA |
| 12 months | 4.96 | 4.44 | 0.52 (−0.12 to 1.16) | 6.12 (−1.36 to 14.13) |
| 30 months | 8.31 | 6.34 | 1.97 (0.48–3.59) | 16.28 (3.84–30.97) |
| **ESCC, *n*** | 274 | 274 | NA | NA |
| 12 months | 4.89 | 4.57 | 0.32 (−0.22 to 0.86) | 3.74 (−2.51 to 10.26) |
| 30 months | 7.99 | 6.60 | 1.39 (0.14–2.65) | 11.02 (1.12–21.76) |
| **ESCC PD-L1 CPS ≥10, *n*** | 143 | 143 | NA | NA |
| 12 months | 8.03 | 6.71 | 1.32 (0.71–2.04) | 16.11 (8.33–26.12) |
| 30 months | 13.02 | 8.89 | 4.13 (2.70–5.76) | 35.69 (21.28–54.58) |

*AE* adverse event, *CPS* combined positive score, *ESCC* esophageal squamous cell carcinoma, *ITT* intention to treat, *NA* not applicable, *PD-L1* programmed cell death ligand 1.
